# Analyzing the impact of psychological capital and work pressure on employee job engagement and safety behavior

**DOI:** 10.3389/fpubh.2022.1086843

**Published:** 2022-12-22

**Authors:** Muhammad Shoaib Saleem, Ahmad Shahrul Nizam Bin Isha, Chizubem Benson, Maheen Iqbal Awan, Gehad Mohammed Ahmed Naji, Yuzana Binti Yusop

**Affiliations:** ^1^Management and Humanities Department, University of Technology PETRONAS, Perak, Malaysia; ^2^Department of Occupational Safety and Health, European University Cyprus, Nicosia, Cyprus; ^3^Faculty of Medicine, Sultan Zainal Abidin University, Kuala Terengganu, Malaysia

**Keywords:** safety compliance, safety participation, work engagement, work pressure, psychological capital, construction industry

## Abstract

**Introduction:**

Buildings and infrastructure are the primary focus of the construction industry, which also includes related activities such as design, planning, demolition, renovation, maintenance, and repair. Safety performance is crucial to the industry's ability to work effectively in spite of hazardous conditions on the job site during any given project. Improving construction workers' safety performance in Malaysia requires an in-depth examination of the interplay between workers' psychological capital, work pressure, employee engagement, and safety participation.

**Methods:**

Administrative and field workers from different divisions across Malaysia's six regions were randomly sampled to collect data for this study. The workers were given a total of 500 questionnaires, of which 345 were returned to the team of researchers. Based on the data analysis, there is an effective interaction between the factors tested toward safety performance.

**Results:**

According to findings, psychological capital positively and significantly affected workers' work engagement. Also, work engagement greatly impacted both workers' safety performance outcomes. Also, as expected, worker pressure significantly and negatively affected workers' safety performance.

**Discussion:**

Insights gained from this research have helped us better organize work and involve employees in safety activities/policies to boost workplace safety performance. The study also suggested that firms should reduce their employees' workloads because doing so would not lower their Psychological Capital but would instead fortify them to better carry out their duties in a risk-free manner.

## Introduction

Buildings and other types of infrastructure are the primary focus of the construction industry's primary activities, which include construction, development, demolition, renovation, maintenance, and repair. However, in recent years there has been a discernible increase in the number of accidents that take place on the job, and many particularly hazardous events have particularly hard hit the construction industry. Accidents in the construction industry that lead to worker injuries and illnesses include falling objects, slipping, tripping, falling from heights, collapsing trenches, and scaffolder failures. These are just some accidents that can occur (1–4). Additionally, the construction industry experienced an injury rate of 2.8 per 100,000 workers in 2019, accounting for more than 1,102 fatalities resulting from workplace accidents (5). In addition, the case of occupational accidents in the Malaysian construction industry is not exceptional; a total of 616 fatalities were reported between the years of 2015 and 2020 (6–9).

Despite substantial progress in safety in the construction industry, there is still room for improvement in the challenge to eliminate the occurrence of a potential accident with unanticipated consequences for the facilities and the human population (10, 11). Furthermore, many industrial safety researchers of accident investigation believe that a single factor never causes an accident. Instead, major industrial incidents are caused by operational, behavioral, and technological factors. Having said that, some of these characteristics are more prevalent than others. In addition, a report on accident investigations conducted by the International Labor Organization (ILO) identifies occurrence factors such as safety compliance, work engagement, psychological capital, work pressure, and participation as factors that influence one another and lead to the potential cause of accidents in the construction industry (12).

Prior literature upholds that certain factors specific to the construction industry can have detrimental impact on one's performance, as no individual is entirely immune to the environmental stress in which they operate (13). For construction workers, stress may include a heavy workload, time pressure, shift-based work, stress from extreme heat, and an unfavorable working environment [(13, 14), p. 201]. Since stress may derail one's attention at work, leading them toward unengaged and unsafe behaviors, this necessitates a scholarly inquiry, where other contextual variables are added to strengthen overall safety.

In addition, a factor such as safety compliance is “the degree or extent to which individuals or workers comply with safety standards, rules, terms and conditions, and regulations at the workplace,” as defined by (15–17). In the case of the construction industry, many organizational factors, such as leadership participation, workload, and work pressure, have been explored as possible predictors of safety-compliant behavior, worker involvement, and safety rule clarity. However, some of the studies' similarities concentrate on safety compliance as a potential predictor of compliance explanatory factor, constructed based on workers' expectations of the specific subject (16, 18, 19). Furthermore, compliance safety has prompted significant research into non-compliant behavior. Over the past few years, many researchers have shifted their attention from identifying the circumstances that lead to a lack of safety compliance on the job site to identifying the factors that increase compliance. Studies such as this one have focused a lot of attention on the issue of safety compliance and safety participation (10, 11, 20, 21).

On the other hand, these studies pose significant challenges to safety measures, rarely evaluated over extended periods (22). Furthermore, in the construction industry, safety is highly enforced. All operations at work are governed by rules and regulations that provide a high level of protection and therefore demand strict adherence. However, greater compliance with safety regulations at the workplace can only be achieved through a comprehensive understanding of effective management methods. Actually, the industry had the same problems as every other sector (23). Hence, there is no agreement regarding the most significant compliance safety factor that influences workers' compliance with safety at work. The study's aims are as follows:

- To determine the factors that influence safety compliance and safety participation in the construction industry.
- To explore the predictive performance of safety factors on safety compliance and safety participation behaviors in accident and risk prevention.

If these goals are accomplished, there is a possibility that the level of safety performance in the construction industry will improve. Following that, the proposed method assists in the identification of critical risks, and increasing safety will reduce the global threat of major hazard incidents, particularly in the Malaysian construction industry. The remaining parts of this paper are broken down into the following sections: Section Literature review presents the theoretical framework, and Section Methodology details the methodology. In Section Results, we will go over the results, in Section Discussion, we will review the discussion, and in Section Conclusion, we will look at conclusions.

## Theoretical background

An accident is an unplanned event with unfavorable consequences (24). There is also the possibility that this could be due to overlooked or unidentified risks. Construction-related accidents have occurred, however, because some of these compliance criteria do not follow workplace safety compliance (16). Out of many reasons, ne of the problem associated with safety compliance is the procedural attentional or non-attentional slips (25, 26).

According to Griffin and Neal (15), Safety compliance is one of two components of the phrase “safety behavior,” which is more widely used in safety performance system research; the other component is “safety participation.” Safety participation is workers' ability to support colleagues, discuss safety issues, and provide safety recommendations to improve workplace safety. The key safety duties that individuals must perform to stay up with and maintain workplace safety standards are referred to as safety compliance. Safety compliance is sometimes defined as “behavior associated with following safety protocols and performing activities safely” (10, 11, 15, 27), but safety participation goes beyond normal call of duty, where individual participate on his/her own, without enforced rules or laws.

**Figure 1:**
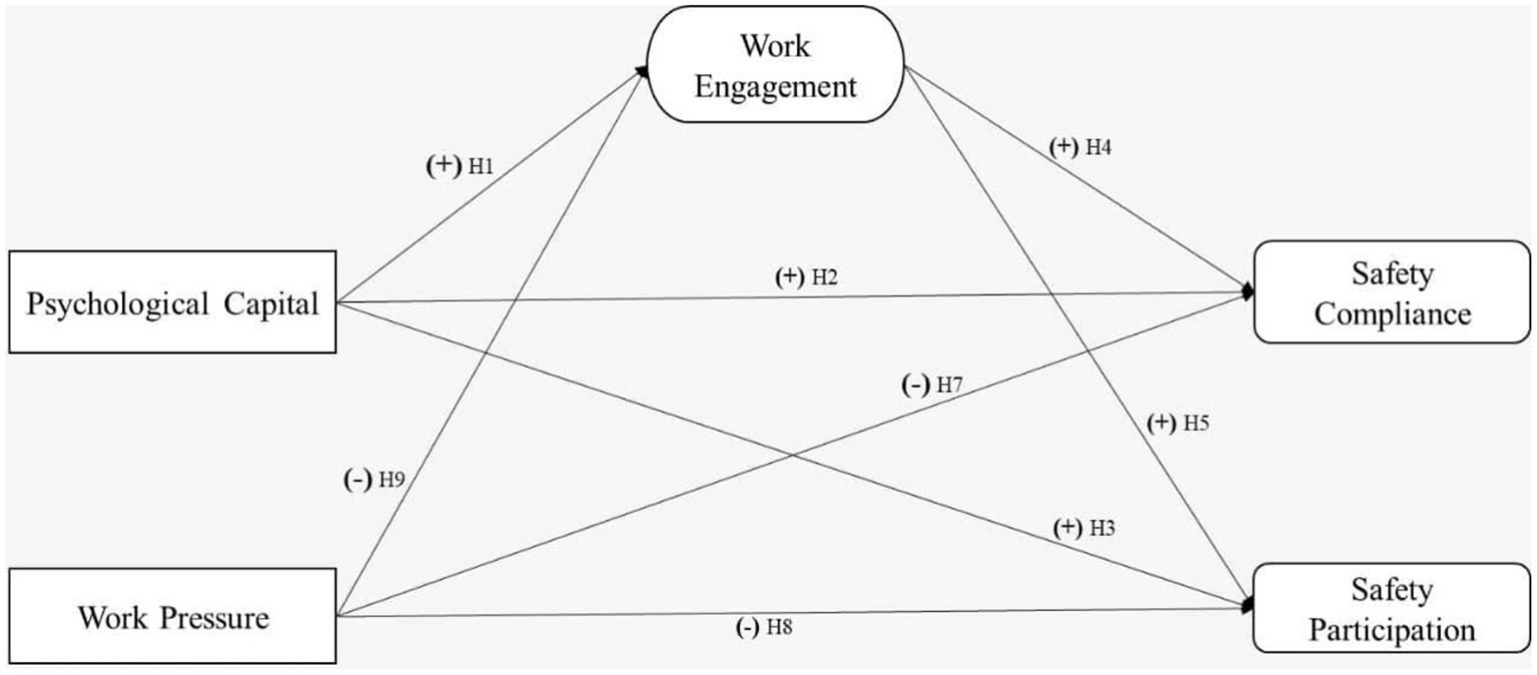
Proposed research framework.

The term “safety compliance” is used to refer to how well employees follow all applicable safety policies, procedures, guidelines, and laws in the workplace (17). Factors such as leadership participation, workload, and work pressure in the construction industry have been investigated as potential predictors of safety compliance behavior (24, 28). In addition, the studies on safety in other industries have identified safety compliance to work in connection to safety competence, safety participation, work engagement, psychological capital, work pressure, and risk (10, 11). Based on the aforesaid discussion, a brief framework of individual safety behavior for the construction industry has been proposed in highlighting safety compliance and safety participation. See Figure 1.

## Literature review

### Psychological capital

Psychological capital was ranked as one of the factors that played a major role in safety performance in the construction industry. However, this factor has to do with individual growth in measuring performance, especially safety compliance (29). About the nature and development of psychological capital, if identified as a personal trait, a personality is also inherited and can be classified as neuroticism, extraversion, openness, agreeableness, or conscientiousness, as an individual's traits are the result of environmental influences (30). It is also expected that when workers possess a higher psychological capital (a personal cognitive resource, one's “positive developmental state”), they are expected to have a higher work-related engagement and enhanced level of personal safety (31). In correspondence to those above, the importance of one's psychological capital to eliminate stress and focus more on engaging behavior is a key personal resource, that can be leveraged (32). According to the (JD-R) Model, psychological capital influences workers' physical and social wellbeing, and work pressure, and excessive workload lead to the decreased safety performance in the construction industry (33). Additionally, individuals who use their psychological capital will attain good safety performance and perform effectively with respect to their work-related engagement (34). See Figure 1, based on the discussion, following hypotheses are formed to be tested. Psychological capital would positively impact Hypothesis 1: Work engagement, Hypothesis 2: Safety compliance, and Hypothesis 3: Safety participation.

### Work engagement

Work engagement is an individual's motivating, a productive work-related condition that includes vigor, dedication, and absorption (35). Engaged personnel have greater enthusiasm and affection for their work, and they are usually so engrossed in their work that time rushes by unnoticeably (36, 37). Workers that are engaged in their work have a strong absorption in their work, like challenges, and have a high mental resilience, which allows them to overcome obstacles while still loving their work. According to the findings of the study, the medical staff was able to provide quality treatment to patients, and those patients reported feeling happier after having interactions with engaged professionals. This led to an increase in overall job effectiveness as well as an improvement in the quality of care provided (38). Work involvement also leads to better interpersonal ties among workers, which produces a healthy work environment.

Work engagement and enhanced interpersonal ties are believed to develop a proactive attitude among workers, leading to greater organizational performance. Work engagement has been classified into three subcategories such as trait engagement (positive view of life and work), state engagement (feeling of energy absorption and effectiveness), and behavioral engagement (extra-role behavior) (36). We will use a state and behavioral engagement viewpoint to link work engagement to safety performance because safety performance comprises safety compliance and participation. According to Idris and Dollard (39), the positive outcome of organizational citizenship behavior is the major basis for combining work engagement with safety behavior, which enhances the effectiveness of the organization. In addition, employees who have a higher level of interest with their work are more likely to engage in safe behaviors (40).

Additionally, engaged workers are more likely to participate in safety activities because they have higher levels of self-esteem, self-satisfaction, and well-connect with their job or role through vigor, dedication, and absorption. As a result, it is expected of them to go beyond the normal call of duty for the safety of the workplace and exhibit participatory behavior by encouraging others to participate in safety, giving suggestions and views to improve safety, and volunteering in safety-related programs (41). Furthermore, in the construction industry, some prominent acts by workers, such as highlighting possible hazards and reporting small injuries and unsafe conditions, are also classified as work engagement behavior (42). The higher the work engagement, will enhance job resources, such as autonomy, will modify safety compliance (in-role conduct) and safety participation (extra-role behavior). See hypotheses 4, 5, and 6 in Figure 1, work engagement would positively influence safety compliance, safety participation, and psychological capital in the construction industry.

### Safety participation

Safety compliance and accident prevention strategies have been dealt with based on various theoretical approaches, and little consideration has been given to the workforce's potentially positive and proactive role in safety management (43). Traditionally, the worker has been viewed as a passive participant in the dynamics of organizational safety (44). On the other hand, more contemporary models of safety performance and involvement have recognized them as the main characteristic of organization safety performance (45). On the other hand, the term “safety participation” refers to a worker's capacity to participate in and comply with workplace safety activities while simultaneously carrying out their regular jobs. In addition, participating in safety means offering suggestions and receiving feedback, motivating others to learn, behave, and perform in a safe manner, actively learning and participating in safety training, bringing attention to potential safety-related problems, and serving as a steward in one's place of employment (46). Furthermore, it is embedded with the responsibilities and duties of a safety officer to encourage workers to participate and show their level of understanding about safety, which in turn will facilitate safety performance (15). Please refer to Figure 1, in which the role of safety participation and safety compliance for the construction industry has been hypothesized.

### Work pressure

Work pressure was discovered to be an important factor influencing safety compliance in the research (47). Workplace stress is frequently mentioned in the context of workplace health issues (48, 49). Safety-related behavior, such as safety compliance, has been investigated using the model (50). According to Nahrgang et al. (51), high work demands and insufficient work resources were negatively associated with safety compliance. In a separate study (52), they discovered a positive relationship between routine violations and work demands (*via* work stress), as well as a negative relationship between work resources and frequent violations (*via* commitment). According to research, job resources and safety compliance are linked (53).

In addition, when it comes to estimating the available work resources, the results of work pressure have been inconsistent. In addition, the topic of work pressure has been extensively researched in the field of occupational safety and work-life balance, and its importance in the prevention of accidents cannot be overstated (16, 54). In addition, self-injury reports or workplace accidents and workers' attitudes toward workplace safety may impact safety compliance (55). In conclusion, it is imperative for every sector of the economy that functions in high-risk environments to strike a balance between job pressure and safety precautions in the workplace (56). See Figure 1, in hypotheses 7, 8, and 9, work pressure negatively affects safety compliance, safety participation, and work engagement in the study industry.

## Methodology

### Sample and procedures

This study investigates factors that influence the construction industry's safety performance and explores the interrelationship and predictive performance of safety factors on safety compliance and participation. Data was gathered through a random sample of workers in the Malaysian construction industry, including administrative and field workers in various departments. However, questionnaires were used to collect information such as participants' age, years of experience, educational levels, and other pertinent details essential to the study. Experts in the field of research conducted a validation evaluation of the questionnaires to ensure that they covered or collected all the information needed for the study.

The final questionnaires were delivered to a random sample of 500 workers after all the appropriate assessments, with 28 ongoing construction projects in six provinces of Malaysia. A total of 345 questionnaires were completed and returned to the research team. Participants used a five-point scale to answer all the questions, ranging from strongly disagree to agree. For data analysis, SPSS-21 and SPSS AMOS-21 software were used to evaluate descriptive and reliability statistics, and confirmatory factor analysis (CFA) to examine the Validity of dimensions, including discriminant and convergent validity. Amos-21 was also used for structural equation modeling (SEM) and hypothesis testing.

### Scale utilized

This study used the measurement established by prior research (57) for the PsyCap measurement. There has been other research that has made use of this questionnaire, most notably in the context of the construction sector as well as some different industry contexts [(29), p. 20, (58–62)]. PsyCap consists of four separate sub-dimensions, which are hope (perseverance to reach the goal and alignment when necessary), self-efficacy (belief in one's abilities and expending efforts to succeed), resilience (tendency to bounce back after being adversely affected), and optimism (looking at the bright side through positive attributes). A total of 24 statements were included in the questionnaire, and six items were allotted to each of the sub-dimensions. An illustration of this would be the phrase, “I am comfortable analyzing a long-term problem in order to discover a solution.” “At work, I normally handle unpleasant situations in one way or another,” When things at work are uncertain for me, I typically expect the best,” “There are many routes that can be taken to avoid any difficulty.”

Prior researchers used a scale to measure work engagement (63). This scale is a condensed form of the work engagement scale, which consists of nine individual components. This one-dimensional scale illustrates three facets of employee engagement: vigor (a higher energy level combined with sufficient mental resilience), commitment (inspiration and excitement toward one's work), and absorption (being completely absorbed in one's work; well-connected and engrossed with work throughout). A 5-point Likert scale was utilized for the work engagement scale that we developed. Some of the comments that were used as questions were as follows: “When I am working, time seems to fly by,” “I take pride in the work that I perform,” and “At my job, I feel powerful and vibrant.”

One of the well-known instruments that Neal and Griffin had devised was used to conduct the safety behavior evaluation (5, 64). The cale consists of total six elements, representing different safety behaviors, that includes three statements for safety compliance (obligatory safety behaviors at the workplace, needed formal observation), and three statement for safety participation (extra-role behavior, not essential to perform, but self-generated behavior in the form of contextual performance). Statements such as “I utilize all of the necessary safety equipment to accomplish my job,” “I employ the correct safety procedures for carrying out my job,” and “I assure the highest levels of safety while I carry out my job” are examples of statements that demonstrate compliance with safety regulations. Remarks such as “I support the safety program within the organization” are examples of safety involvement statements. The phrases “I make an extra effort to improve workplace safety” and “I voluntarily carry out duties or activities that promote workplace safety” refer to the same action: making an extra effort to improve workplace safety.

The level of work pressure was determined using a scale with five components (55). The amount of pressure at work is shown through statements such as, “The primary focus of this organization is on production, everything else is secondary;” “If production demands are not met, I may be demoted or endure other unpleasant consequences relating to my employment.” A five-point Likert scale was used to grade everything on the list.

### Data collection and methods

The respondents in this study were employed in the Malaysian construction industry. Because we do not possess a comprehensive list of the population being investigated, we resorted to the non-probability, convenience, and snowball sampling strategies (65). The data collection was finished after 4 months. Hard copies of the instrument were given to those who worked in the following trades: roofing workers, masons, plumbers, tile and brick installers, ironworkers, electricians, pipefitters, and concrete finishers. Responses were completed whenever workers were available, including during break intervals, meal breaks, and other pauses in the workday. During the process of providing their answers, respondents received assistance. The research team received the questionnaires once they had been filled out and submitted. The survey might be finished in 10–15 min. All ethical considerations were kept in mind while undertaking this study i.e., respondents were ensured of their privacy and anonymity, two of the most important aspects of any survey. Before the study began, respondents were asked to sign a consent form, and their participation in the research was optional. From the demographics point of view, 85% respondents were male, and 15% respondents were female. Participants ranged from 25 to 35 years old, with work experience ranging from 6 to 10 years. The participants' educational levels ranged from a professional degree to High school. See Table 1—demographic information of the participants.

**Table 1:** Demographic information of the participants.

**Demographic variables**	**Findings**
Respondents' average age bracket	25–35
Gender	85% Males, 15% Females
Respondents' average working experience	6–10 Years
Respondents education	45% Diploma, 19% high school etc.
Distribution of responses (state-wise)	Penang (11%), Selangor (17%)
	Johor (23%), Perlis (33%)
	Perak (5%), Melaka (11%)

## Results

### Reliability and validity results of the study

A reliability test was carried out to verify the internal consistency of the questionnaire constructs. Cronbach's alpha must be sufficiently high to achieve acceptable response reliability (66). For all five constructs, Cronbach's alpha values varied from 0.868 to 0.949, showing that our findings are reliable. See Tables 2, 3 representing the results of Confirmatory Factor Analysis (CFA), convergent and discriminant validity. Convergent validity refers to the interpretive power of observable variables over latent variables. It might be evaluated using three common indicators, i.e., standardized factor loadings (SFL), construct reliability (CR), and average variance extracted (AVE). Factor loadings of all constructs were well above the cut-off limit, i.e., 0.70, where the standardized factor loading for psychological capital ranged from 0.70 to 0.756, work engagement from 0.70 to 0.76, work pressure from 0.84 to 0.88, and for safety performance 0.794–0.864, hence exhibiting a strong convergent validity.

**Table 2:** Result of convergent and reliability analysis.

**Constructs**	**Cronbach's alpha**	**Composite reliability**	**Average variance extracted (AVE)**
Work pressure	0.934	0.950	0.791
Psychological capital	0.949	0.954	0.552
Safety compliance	0.874	0.922	0.798
Safety participation	0.868	0.919	0.790
Work engagement	0.904	0.922	0.567

**Table 3:** Result of discriminant validity analysis.

**Constructs**	**Work pressure**	**Psychological capital**	**Safety compliance**	**Safety participation**	**Work engagement**
Work pressure	0.889				
Psychological capital	−0.166	0.743			
Safety compliance	−0.381	0.218	0.894		
Safety participation	−0.288	0.207	0.284	0.889	
Work engagement	−0.072	0.741	0.298	0.209	0.753

Results of the concurrent validity indicators that demonstrated an acceptable convergent validity. See Table 2, to describe the discriminant validity of the constructs, there are common indicators like construct reliability (CR > 0.7), standardized factor loadings (SFL > 0.6), and average variance extracted (AVE > 0.5) that were used/compared (67). For discriminant validity, the square root of the average variance extracted (AVE) value was compared with the correlation coefficient of other variables (67). If the outcome value is greater than its correlation coefficient, then the discriminant validity was achieved, and all our constructs met this criterion. See Table 3.

To assess the validity of the measurement model for all variables, we used the subset-item parceling technique for psychological capital and work engagement, since they were taken as a higher-order construct in this study. Through this technique, the aggregation of individual items of a construct into one or more parcel(s) is performed to exhibit a latent variable (68, 69). Furthermore, the method allows researchers to form parcels by adding or averaging items in the desired parcel(s), e.g., if a scale carries eight items, the practitioner can aggregate all items to form one parcel or even two items to form four parcels. Our result indicated a good fit for measurement models (70).

A few of the indices like CMIN (chi-square *X*^2^ /degree of freedom), chi-square *X*^2^, comparative fit index (CFI), root-mean-square error of approximate (RMSEA), normed fit index (NFI), goodness-of-fit index (GFI), adjusted goodness-of-fit index (AGFI), Tacker-Lewis index (TLI) were used (71–73). All the values for each index were well under the criteria. Results for five factors measurement model were (*p*-value = 0.183, RMSEA = 0.018, GFI = 0.958, NFI = 0.972, AGFI = 0.943, CFI = 0.997, TLI = 0.996, Chi-square = 139.123, and CMIN = 1.113, and DF = 125). To summarize the reliability, convergent Validity, and discriminant validity, our study findings support that the internal factor structure of the scales being tested is well-validated and reliable by meeting the convergent and discriminant criterion (74). These data corroborate the instruments' efficacy and predictability in this investigation.

### Structural model and hypothesis testing

The hypothesis model was created and tested using the SEM method to determine the Goodness-of-Fit; we examined whether the results fit the measurement and a structural model. For structural model fit criteria, the primary goal was to determine whether there were any abnormal variables, where all variances were significant with a value >0, standard errors were well under the limit, and all standardized factor loadings were substantial. Our results exhibit strong empirical evidence for the good primary fit of the data (i.e., *p*-value = 0.228, RMSEA = 0.009, GFI = 0.914, NFI = 0.922, AGFI = 0.902, CFI = 0.997, TLI = 0.997 and CMIN = 1.031). All the indexes met the criteria, demonstrating an acceptable overall model fit.

To test the hypothesized model, we employed a 95% confidence interval. We accepted or rejected research hypotheses based on the *p*-value and the nature of effect size (based on presupposed theoretical assumptions). As per the data outcomes, hypothesis−1, psychological capital, work engagement (β = 0.749, *p* < 0.001) was accepted. Unexpectedly, hypothesis−2, psychological capital, safety compliance (β = −0.096, *p* > 0.05), and hypothesis 3, psychological capital, safety performance (β = −0.05, *p* > 0.05), both were rejected based on their insignificant *p*-value. For hypothesis 4 and hypothesis−5, work engagement, safety compliance (β = 0.342, *p* < 0.05), and work engagement, safety participation (β = 0.153, *p* < 0.05), both were accepted. Also, as expected for hypotheses 7 and 8, Work pressure, safety compliance (β = −0.373, *p* < 0.05), and Work Pressure, safety participation (H6; β = −0.269, *p* < 0.05) both were accepted, unexpectedly, hypothesis−9 for Work pressure, work engagement (β = 0.052, *p* > 0.05) was rejected based on its insignificant *p*-value, regarding the overall variance caused in dependent variables by their independent variables; for work engagement, it was 0.549 (54%), for safety compliance 0.216 (21%), and safety participation 0.122 (12%; through their predictors).

### Mediation results

Furthermore, analysis of mediation results indicates that work engagement played a partially mediating role between psychological capital and safety compliance (β = 0.203, *p* < 0.001), as well as between psychological capital and safety participation (β = 0.142, *p* < 0.001), hence hypothesis-6 was accepted. Nonetheless, there was an insignificant mediation effect observed between work pressure, work engagement, and safety performance objective indicators; safety compliance (β = 0.014, *p* > 0.05) and safety participation (β = 0.01, *p* > 0.05). See Table 4 for further explanation results of the hypotheses and correlation amongst all the variables.

**Table 4:** Result of hypothesis testing.

**Variables**	**Effect** **size**	**Sample mean (M)**	**Standard deviation (STDEV)**	***T*-statistics**	***p*-values**	**Hypothesis** **supported**
Psychological capital -> Work engagement	0.749	0.751	0.048	15.537	0	Yes
Work engagement -> Safety compliance	0.342	0.359	0.087	3.938	0	Yes
Work engagement -> Safety participation	0.153	0.162	0.067	2.286	0.022	Yes
Work pressure–> Work engagement	0.052	0.053	0.054	0.963	0.335	No
Psychological capital -> Safety participation	0.05	0.044	0.069	0.717	0.473	No
Psychological capital -> Safety compliance	−0.096	−0.109	0.085	1.127	0.26	No
Work pressure -> Safety participation	−0.269	−0.266	0.048	5.634	0	Yes
Work pressure -> Safety compliance	−0.373	−0.369	0.048	7.818	0	Yes

## Discussion

The purpose of this research was to improve our understanding of the factor structure of work engagement in the construction industry by placing it in context with other significant environmental factors and human traits that may be used to make predictions about safety performance. On the other hand, the findings of this study have evaluated the influence of psychological capital as an indication of workers' performance and job engagement, and they viewed work engagement as a mediating variable between psychological capital and safety performance.

Secondly, we also assessed the impact of work pressure on work engagement and safety performance indicators. Study findings highlighted that psychological capital had a significant and positive impact on the work engagement of construction workers. However, it was unexpected to observe that psychological capital was found to have a non-significant association with safety performance objective indicators, which was against our expectations and in contrast with prior research (31).

The workforce in the construction business is anticipated to experience workload and work pressure with strict timetables, which may cause workers to take additional risks and adapt risky approaches to finish operations more rapidly (32). Workers might not feel as self-sufficient as they should in order to demonstrate the safety compliance and participation behaviors that are expected of them. This is another possibility. The majority of people who work in construction come from rural areas, and as a result, they struggle with communication issues, feelings of alienation from their families, and the acquisition of new skills. These challenges can lead them to feel overburdened and cause them to ignore safety-related regulations and policies (33).

Further, work engagement mediated between psychological capital and safety performance in both dimensions, which is in harmony with the (11, 27, 32). Our research findings supported the notion that if positives at the workplace are on the higher side, they may increase the worker's motivational state, and in our case, it is work engagement. Therefore, our results are in harmony with the job-demand-resource-model (33), which advocates the balance between negatives and positives at the workplace in terms of resources for employees. It would be helpful for organizations to strengthen the psychological capital of their workforce so that they have more psychological resources available to them, which in turn will help them to be more engaged and a safe employee.

Also, as expected, work pressure showed a significant negative association with safety compliance and participation, meeting our study assumptions. In contrast, the direct effect of work pressure on work engagement was found non-significant. Further, no mediating effect was observed between work pressure, work engagement, and safety performance, which was contradictory to the expectations (11, 34, 51, 75). In our case, employees' excessive participation in work may be a possible justification for this situation, as engagement at work goes beyond the normal call of duty (43). An individual's overpowering desire to be more productive at work through increased involvement may cause them to disregard their own personal safety as well as the safety of those around them in order to achieve their professional goals. Our findings were counterintuitive to those of earlier research, which suggested that engaged workers were more likely to be safe workers. Since our findings were inconsistent, it is clear that additional empirical data is required to support this hypothesis. Instead of relying on activities that are indirect in nature, organizations need to design solutions that establish a direct link between work pressure and the safety phenomena (45, 46).

Our findings also supported that work pressure has a detrimental impact on safety performance, which is consistent with previous research (10, 11, 28). Among the various factors that may have alleviated this negative relationship could be fear of being laid off, management prioritization of production over safety, supervisory or management push for output, lean manufacturing, work overload, etc. Other factors such as working hours, delays in working schedule, operating speed and shift timings might also exacerbate the work pressure (24), which may, in turn, force construction workers to perform their tasks unsafety.

Additionally, some of the reasons associated between work pressure and safety behavior could be ineffective workforce management and rework of the construction industry (16, 18, 19). Such factors are expected to escalate the stress level for construction workers, reducing their production efficiency and ultimately creating an environment where occupational health and safety may be compromised. It is clear from the findings that firms must strike a balance between productivity and safety, where workers are pushed to productivity while remaining safe. According to Hanna and Markham and Sinclair et al. [(4), p. 20, (21)], health and safety for construction can be better operationalized if the overall structural constraints of this industry are realized, as it will help organizations to come up with feasible changes that can benefit mutually.

## Conclusion

The ability of those in the construction sector to function efficiently in hazardous environments is mostly dependent on safety. As a result of our research, we came to the conclusion that psychological capital is not associated with either safety compliance or safety participation. This result ran counter to our expectations, which suggested that the availability of sufficient positive psychological resources might play a role in the generation of safety-related behaviors such as compliance and participation. It is possible that the lack of these psychological resources for workforce members in the construction industry is one of the contributing elements to the construction industry's failure to comply with safety rules and other predictable scenarios. It was also found that having positive psychological resources would help employees act in a way that makes them feel engaged, which will help them act in a way that is safe. Pressure at work was also found to affect safety performance in the construction industry in a big way and in a bad way. Lastly, we found that work engagement had a positive effect on both safety behaviors, which also fit with what we thought our study would find.

The most effective approach for increasing worker compliance and balancing their psychological capital is to constantly motivate workers to adhere to safety participation. Good work engagement will minimize work pressure in industrial construction projects. Furthermore, applying a substantial understanding of suitable management methods for compliance improvements with safety is important. Encouraging workers to observe safety compliance would lower safety risk and accidents in the construction industry. The study's findings have contributed to our understanding of improving and ensuring workplace safety compliance by encouraging optimal work organization and worker participation in safety. The findings of this study have a number of important implications for the construction industry. One of the most important of these is the need to search for the optimal position in which workers are not subjected to an excessive amount of work pressure and are instead kept engaged in such a way that they naturally operate in a safer manner. Through trainings and individualized sessions, the construction sector can also contribute to the improvement of its workforce's hope, sense of effectiveness, resiliency, and optimism. Because of this, they will be better able to integrate themselves into their work environment, which will ultimately enable them to accomplish their duties in a manner that is far safer. In addition, the study recommended that the construction sector limit the amount of work pressure, which has the potential to deplete the workers' psychological capacity, and increase worker strength to ensure that workers follow safety performance and safety compliance in their duties.

## Research limitations

Researchers frequently use cross-sectional data, but longitudinal data may 1 day lead to stronger and more reliable results. Also, we used self-reported assessments for all study variables, which could have led to overestimation of PsyCap, work pressure, engagement behavior, and the overall safe-performance by participants. Our research work, has only been validated inside the Malaysian cultural setting, and it has to be expanded. Furthermore, the majority of our respondents were workers, most of whom lacked a higher level education. Consequently, more could be learned through study with a larger sample size of people from various backgrounds. Our respondents were workers, a transient workforce whose employment is often tied to specific projects. As a result, the findings may not be generalizable to other professional populations. Potentially different findings could be obtained from applying this methodology to other potentially dangerous occupations where workers are retained for extended periods of time. Another limitation of our study is that most people working in the construction industry are men, thus we have a skewed gender ratio among our respondents. It's possible that the outcomes might change if we included just sectors with an optimal demographic mix. In future, researchers should assess psychological capital as a team phenomenon, where it could be related with other safety-related individualistic behaviors.

## Data availability statement

The raw data supporting the conclusions of this article will be made available by the authors, without undue reservation.

## Author contributions

Idea inception and execution were done by MS and GN, leading by content analysis and writing. AI, CB, and YY further refined, revised, and reviewed the overall manuscript writing. All authors have read and approved the final manuscript.
